# Community surveillance after detection of poliovirus in the environment in London, United Kingdom, October 2022 to April 2023

**DOI:** 10.2807/1560-7917.ES.2025.30.16.2500025

**Published:** 2025-04-24

**Authors:** Thomas Rowland, Robin Gopal, Monika Patel, Cristina Celma, Colin NJ Campbell, Nicholas Machin, Scott Taylor, Shauni-lea Graham, Kathryn Harris, Spiro Pereira, Vanessa Saliba, Maria Zambon

**Affiliations:** 1 Virus Reference Division, UK Health Security Agency, London, United Kingdom; 2 Clinical Academic Graduate School, University of Oxford, Oxford, United Kingdom; 3 Polio Reference Service, Virus Reference Division, UK Health Security Agency, London, United Kingdom; 4 Enteric Viruses Unit, Virus Reference Division, UK Health Security Agency, London, United Kingdom; 5 Immunisation & Vaccine-Preventable Diseases Division, UK Health Security Agency, London, United Kingdom; 6 UKHSA and Manchester University Hospitals NHS Trust Medical Microbiology Partnership, Manchester, United Kingdom; 7 Virology, Barts Health NHS Trust, London, United Kingdom

**Keywords:** poliovirus, VDPV2, surveillance, outbreak investigation

## Abstract

**Background:**

Vaccine-derived polioviruses (VDPVs) continue to circulate internationally, causing sporadic cases and outbreaks of paralytic polio in countries certified as polio-free. In 2022, sustained detection of type 2 VDPVs was reported in environmental surveillance samples collected from London. Genetic mutations indicative of loss of attenuation of virulence were observed, consistent with community transmission events over several months.

**Aim:**

We aimed to determine the extent of geographical spread of transmission in an area of environmental poliovirus detection.

**Methods:**

We implemented an opportunistic, cross-sectional survey in areas where environmental surveillance indicated sustained VDPV transmission between October 2022 and April 2023. Residual stool samples taken from children < 16 years presenting to primary or secondary healthcare were examined for enteroviruses, including poliovirus. Methods for poliovirus detection recommended by the World Health Organization, including virus isolation in cell culture, PCR and molecular characterisation, were applied to residual stool material on a daily basis with real-time clinical reporting.

**Results:**

We examined 1,251 stool samples from 1,051 children presenting to healthcare with illness over a 6-month period. A range of enteroviruses from groups A, B and C were found, but no poliovirus was detected. Documented polio vaccination coverage was high, between 95% and 98% in under 5-year-olds.

**Conclusion:**

Poliovirus was not widespread in the area of environmental poliovirus isolation. Opportunistic poliovirus testing of residual stool samples taken from children seeking healthcare was feasible and can be implemented rapidly in areas where poliovirus circulation is suspected, although untargeted sampling may not adequately capture populations at highest risk.

**Key public health message**

**What did you want to address in this study and why?**

A person infected with poliovirus may be infectious without having any symptoms. Therefore, when poliovirus is detected in sewage, the World Health Organization recommends searching for undetected cases in the local area. After poliovirus was persistently detected in London sewage in 2022, we tested stool from children in that area to find infected individuals who may have been contributing to silent transmission.

**What have we learnt from this study?**

We collected samples from children undergoing routine clinical investigations within the area where the poliovirus was detected. None of the 1,251 samples analysed over a 6-month period were positive for poliovirus. By testing stools that had already been collected for other reasons, we were able to look for infected individuals more quickly and with fewer resources than if we also had to establish new clinical pathways for taking samples.

**What are the implications of your findings for public health?**

When poliovirus is detected in sewage but we do not know of any symptomatic individuals, testing stools collected for other reasons in the local area is a practical approach to looking for cases. As sewage surveillance becomes available in more countries, analysis of leftover stool samples collected for other reasons could be adopted more widely to support enhanced polio surveillance in outbreaks.

## Introduction

Poliovirus was historically a major cause of morbidity and mortality. Since the introduction of the inactivated polio vaccine (IPV) in 1955 and oral polio vaccine (OPV) in 1959, two of the three wildtype strains (PV2 and PV3) have been eradicated, with most countries now certified as polio-free. Each of the three attenuated Sabin virus strains (Sabin 1,2,3) in OPV can mutate to virulent paralysis-causing strains. Such strains have the potential to circulate for prolonged periods in susceptible communities, described as circulating vaccine-derived polioviruses (cVDPVs). The UK switched from the live OPV, which prevents transmission as well as providing excellent protection from paralysis, to IPV in 2004 [1], 20 years after the last case of polio in the United Kingdom (UK).

Globally, OPV remains an important tool to achieving polio eradication. Despite the withdrawal of the Sabin 2 vaccine component from the trivalent OPV in 2016, there has been continuing use of monovalent OPV2 vaccine in some parts of the world and a sustained rise in the number of cVDPV2 cases internationally since, including sporadic imported cases in previously polio-free countries. During 2023 and 2024, the World Health Organisation (WHO) reported more than 70 cVDPV2 emergences from multiple countries and has continued to maintain the status of polio transmission as a Public Health Emergency of International Concern [2-4]. Since September 2024, poliovirus has been detected through routine surveillance of wastewater systems in five countries in the WHO European Region (Finland, Germany, Poland, Spain and the UK) [5]. While no clinical cases associated with these detections have been identified to date, the presence of the virus in the community emphasises the ongoing risk poliovirus poses to countries everywhere [6]. In countries where polio eradication has been certified, maintaining certification requires high poliovirus vaccination rates in all communities, mainly using IPV, with surveillance for early detection of imported infections. The Global Polio Eradication Initiative (GPEI) recommends a mix of acute flaccid paralysis (AFP) clinical and laboratory surveillance alongside environmental surveillance as part of routine national surveillance programmes [7].

Routine environmental surveillance for poliovirus has been operational in two UK cities, London and Glasgow, since 2014. Polioviruses have been detected sporadically in wastewater. Between February and November 2022 there was a large increase in the number of detections, with more than 100 different infectious poliovirus isolates obtained from sewage samples collected from areas of North and East London [8]. Genetic analysis identified these as derived from Sabin type 2 vaccine strains with an accumulation of mutations consistent with ongoing circulation in the community. The WHO declared this virus a cVDPV2 in May 2022. The UK isolates were genetically linked to poliovirus isolates obtained in the United States (US) and Israel in 2022 [9,10].

The WHO guidance recommends determination of the geographical extent and assessment of the risk of further transmission using a stool survey [2]. We rapidly initiated a residual stool sample survey in areas of environmental detection. This was part of a wider response by the UK Health Security Agency (UKHSA) that included intensified clinical and laboratory surveillance, considerably expanding environmental surveillance across London and nationally, a national IPV catch-up campaign and a targeted IPV booster campaign in London [11].

Our objectives were to identify the extent of geographical spread of transmission in children <16 years in an area of environmental poliovirus detection through opportunistic testing of stool samples.

## Methods

### Sample collection methods

**Figure 1:**
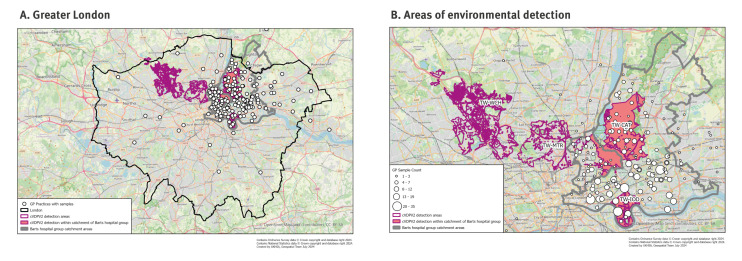
Sewage catchment areas with cVDPV2 detection, catchment area for faecal testing service for general practices, and local hospitals and areas where cVDPV2 was detected, London, October 2022–April 2023 (n = 1,019 individuals)

We performed a cross-sectional study of residual stools from children younger than 16 years presenting with any type of illness within a London hospital group over a 6-month period, from October 2022 to April 2023. The hospital group comprised five major hospitals in an area of North and East London serving a population of ca 1.2 million of all ages, approximately co-terminus with four local authority areas in Greater London: Hackney, Newham, Tower Hamlets and Waltham Forest. Here, 25.0% of the local population are younger than 16 years, compared with 23.9% in Greater London. The pathology service for the hospital group is consolidated on a single hospital site, facilitating acquisition of residual stool samples on a daily basis. The residential catchment area of this hospital group overlapped areas of collection for wastewater treatment works (TW) with the highest levels of persistent detection of cVDPV2s (TW-CAT and TW-IOD) (Figure 1A) and an adjacent area with lower detection rates served by TW-WHI, TW-GCR and TW-FSR (Figure 1B).

cVDPV: circulating vaccine-derived poliovirus; GP: general practitioner.

Note that some samples were taken from individuals outside of the Barts laboratory catchment areas, including some from individuals outside of London (panel B, not all shown). These are likely to represent samples taken from inpatients at one of the hospitals within the catchment area for Barts laboratory who are registered at a GP practice outside of the Barts laboratory catchment area.

### Sample processing

**Figure 2:**
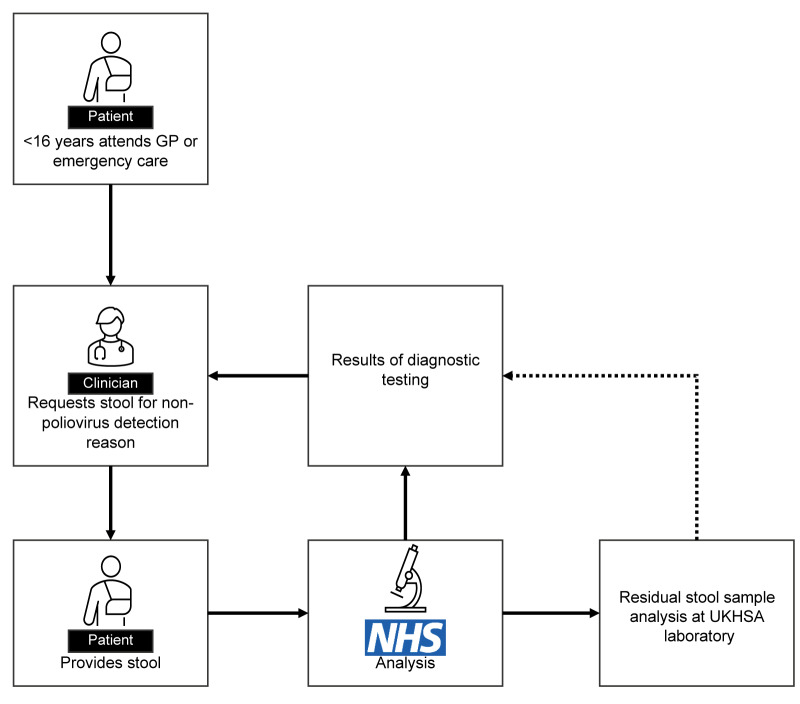
Schematic of sample flow, poliovirus surveillance in residual stool samples, London, October 2022–April 2023

GP: general practitioner; UKHSA: United Kingdom Health Security Agency.

Stool survey #1 ran from 1 October to 30 November 2022 and stool survey #2 from 1 January to 26 April 2023. Sample collection was paused during December, to mitigate the impact of the investigation on the local pathology service through the holiday period. Throughout both surveys, saline suspensions from residual stool samples were created on a daily basis and tested at UKHSA by pan-enterovirus PCR [12]. Samples testing positive were sequenced in the VP1 gene to provide a genotype [13]. The PCR results from both surveys were returned to the primary clinicians in compliance with requirements of the ethical approval of the study protocol (Figure 2). During Stool survey #2, residual stool samples were also tested by virus isolation following the WHO Global Poliovirus Laboratory Network surveillance protocol [14]. Absence of poliovirus growth in isolation cultures was also confirmed by PCR. Additional details on virus isolation are appended in the Supplement.

Faecal samples were collected as part of routine clinical investigations unrelated to the study arranged by general practitioners in the community and hospital specialists on inpatients and outpatients. Where there was adequate remaining sample following routine testing, it was transferred to either a regional UKHSA laboratory or, in 2023, directly to the national poliovirus laboratory at UKHSA Colindale. The regional laboratory tested solely for enterovirus, with positive samples sent on to the UKHSA national laboratory for further typing. Had poliovirus been isolated at the UKHSA national laboratory (not shown), the local health protection team would have been involved to notify the patient who provided the sample and to initiate an outbreak investigation.

### Data linkage and analysis

Ethnic group was self-reported to the patient’s primary care provider and extracted from their vaccination record. We obtained vaccination status of individuals by direct matching of the NHS number of children providing a stool sample with North East London Child Health Information System database. Local vaccination data were extracted from UKHSA’s Cover of Vaccination Evaluated Rapidly (COVER) programme [15]. We considered a child to have completed a primary course of vaccination if they had received three doses of the diphtheria, tetanus and pertussis/inactivated polio vaccine/*Haemophilus influenzae* type b/hepatitis B (DTaP/IPV/Hib/Hep B) vaccine, and received at least one booster if they had received four or more doses. Data analysis was performed using R (v4.2.2, ‘Innocent and Trusting’) in R Studio (2023.09.1, ‘Desert Sunflower’). Geospatial analysis was performed using geographical information systems analysis with ESRI ArcGIS software (Desktop 10.8.2 and ArcGIS Pro 3.3.0) was used to map the locations of the primary care providers (general practitioners (GP)) who requested the stool sample. This was done by linking the postcode of the GP to the Office National Statistics Postcode Directory, through geocoding. We then joined these points to the available water treatment catchment boundaries, as well as the Barts hospital Group catchment area, and calculated the total number of samples per catchment area. Both GP locations and catchment areas were mapped (Figure 1).

## Results

**Figure 3:**
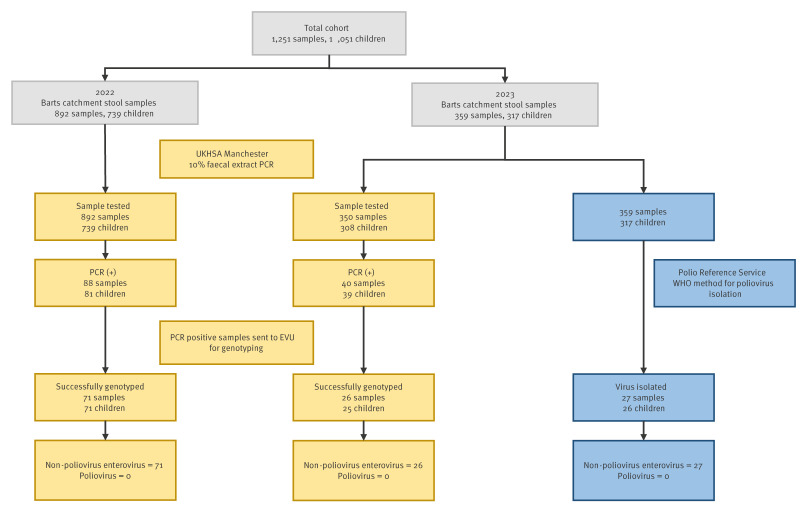
Sample flow, poliovirus surveillance in residual stool samples, London, October 2022–April 2023 (n = 1,251)

EVU: Enteric Virus Unit; UKHSA: United Kingdom Health Security Agency; WHO: World Health Organization.

Between the two surveys conducted in 2022 and 2023, a total of 1,251 samples (892 in survey #1 and 359 in survey #2) were collected from 1,051 children (739 in survey #1 and 317 in survey #2) (Figure 3). The residual sample survey protocol collected all samples, including occasional multiple samples from the same child. Five children provided samples in both surveys. Multiple samplings of the same child were driven by the original clinical requesters.

A total of 1,251 samples from 1,051 children were collected across two surveys: stool survey #1 in 2022 and stool survey #2 in 2023. Many children provided more than one sample within the same survey. Five children provided samples to both stool survey #1 and stool survey #2, and are counted in both as a result.

The median age of children at their first sampling was 5.4 years (5.7 in survey #1 and 5.3 in survey #2) and 580 (55%) were boys (393 (53%) in survey #1 and 189 (60%) in survey #2). The majority of children with this information available (n = 447; 57%) were from the ‘Asian or Asian British’ ethnic group. The next largest ethnic groups were ‘White’ (n = 122; 14%) and ‘Black, Black British, Caribbean or African’ (n = 101; 12%). Compared with the local demographic data, ‘Asian or Asian British’ children were overrepresented (57% vs 39%) while ‘White’ and ‘Black, Black British, Caribbean or African’ were underrepresented (14% vs 28% and 12% vs 17% respectively) (Table 1) [21]. Of children whose GP was known, 978 (93%) were registered within the catchment area of the hospital group study site, which contains two of the four wastewater catchment areas with cVDPV2 detection (Figure 1).

**Table 1:** Demographic data for children sampled for poliovirus presenting to a healthcare provider within the catchment area of London hospital group, London, October 2022–April 2023 (n = 1,051)

Characteristic	Study residual stool survey						UK Census data (under 16 years)				
	Overall (n = 1,051)		Stool survey #1 (n = 739)		Stool survey #2 (n = 317)		Hackney, Newham, Tower Hamlets, Waltham Forest		Greater London		
	n	%	n	%	n	%	n	%	n	%	
Median age in years (IQR)	5.5 (2.2–10.3)		5.7 (1.7–10.6)		5.3 (2.5–9.3)		NA				
Sex											
Female	469	45	344	47	128	40	130,305	49	928,880		49
Male	580	55	393	53	189	60	134,370	51	969,160		51
Not known	2	NA	2	NA	0	NA	NA				
Ethnic group											
Asian or Asian British	477	57	344	56	136	59	102,855	39	436,460		23
Black, Black British, Caribbean or African	101	12	73	12	28	12	45,960	17	320,065		17
Mixed or multiple ethnic groups	79	9	58	10	22	10	26,920	10	222,345		12
White	122	15	90	15	32	14	74,100	28	791,000		42
Other ethnic group	61	7	47	8	14	6	15,790	6	128,265		7
Not known	211	NA	127	NA	85	NA	NA				

IQR: interquartile range; NA: not applicable; UK: United Kingdom

The table shows the summary statistics for age, sex and ethnic group overall and in the 2022 and 2023 surveys, and for sex and ethnic group in the local population. Age is at the time of sampling; where individuals were sampled more than once, the age at sampling was taken from a randomly selected sample. Ethnic group was taken from the NHS demographic database (‘NHS Spine’) and summarised into Office National Statistics ethnicity categories. Note that five individuals appear in both surveys and so the overall total does not match the sum of the individual surveys. Comparison is made to 2021 UK census data for people younger than 18 years in the London Boroughs of Hackney, Newham, Tower Hamlets and Waltham Forest, which geographically closely match the catchment area identified in Figure 1, and to Greater London as a whole [21].

Vaccination records were available for 1,000 of 1,051 (95.1%) individuals. Documented polio vaccination coverage in our cohort was high: 124 (95%) 1-year-olds, 81 (94%) 2-year-olds and 61 (98%) 5-year-olds had completed their primary polio vaccination course (Table 2). Of those older than 5 years, 510 (97%) had completed their primary course (data not shown). This was higher than the contemporaneous average vaccination coverage in the local area, taken as the four local authorities including and overlapping the hospital catchment areas. In these administrative areas, 85% of 1-year-olds, 84% of 2-year-olds and 87% of 5-year-olds had completed their primary polio vaccination course at the time of survey #1.

**Table 2:** Polio vaccination primary course completion among 1-year olds, 2-year-olds and 5-year-olds London, October 2022–April 2023 (n = 291)

Rate of primary vaccination course completion	Study						January–March 2023 COVER data			
	Overall (n = 291)		Stool survey #1 (n = 186)		Stool survey #2 (n = 107)		Hackney, Newham, Tower Hamlets, Waltham Forest		Greater London	
	n	%	n	%	n	%	n	%	n	%
1-year-old (n = 134)										
Completed	124	95	83	93	42	100	3,628	85	22,756	87
Incomplete	6	5	6	7	0	0	646	15	3,394	13
Not known	4	NA	3	NA	1	NA	NA			
2-year-old (n = 91)										
Completed	81	94	50	93	31	97	3,569	84	23,126	89
Incomplete	5	6	4	7	1	3	670	16	2,990	11
Not known	5	NA	2	NA	3	NA	NA			
5-year-old (n = 66)										
Completed	61	98	35	97	27	100	3,743	87	25,061	88
Incomplete	1	2	1	3	0	0	564	13	3,313	12
Not known	4	NA	2	NA	2	NA	NA			

NA: not applicable; UKHSA: United Kingdom Health Security Agency.

The schedule for the polio vaccination primary course in the United Kingdom is with the 6-in-1 vaccine at ages 8 weeks, 12 weeks and 16 weeks. This table shows the rates of completion of this primary course (three or more doses of polio vaccine given) among those aged 12–24 months, 24–36 months and 60–72 months of age. Where individuals provided more than one sample, a single sample’s date of sampling was randomly selected for calculation of the vaccination status. Note two of the children included in this Table provided samples in both surveys, and so the overall total may differ from the total of the two surveys. Comparison is made to the aggregate vaccination coverage across the London Boroughs of Hackney, Newham, Tower Hamlets and Waltham Forest which geographically closely match the catchment area identified in Figure 1, and to Greater London as a whole. London vaccination data come from UKHSA’s Cover of vaccination evaluated rapidly (COVER) programme 2022 to 2023 quarterly data for January to March 2023 [15].

No poliovirus was detected in either stool survey. Enterovirus was detected by PCR in 128 samples from 120 children. Of these, 97 samples were successfully genotyped and in each a specific non-poliovirus enterovirus (NPEV) was identified, confirmed by sequence analysis. Unambiguous sequence identity could not be confirmed in the remaining samples, but poliovirus was definitively excluded. In addition, in survey #2, 359 samples from 317 children were used to attempt viral isolation. No poliovirus was isolated, but a non-poliovirus enterovirus was isolated in 27 samples from 26 children.

## Discussion

This study began in 2022 shortly after sustained environmental isolation of VDPV2 was confirmed in London, in the absence of any clinical case of polio. An opportunistic stool survey was implemented rapidly as a response measure to search for clinically inapparent poliovirus infection, establish the force of infection and inform the scale of the public health response to the detection of VDPV2 in London. Following the detection of poliovirus, local health authorities are advised to search for linked AFP cases or evidence of clinically silent community transmission [2]. This requires enhanced laboratory and environmental surveillance around the site of any poliovirus detection and detailed follow-up investigations and contact tracing of any cases. Control measures such as an emergency immunisation programme may also be required. The only prior report of a stool survey initiated in response to poliovirus detection in environmental samples with no associated clinical cases was in Israel in 2013 [16].

We chose to conduct an opportunistic survey of the local child population. The stool surveys ran for 6 months from October 2022. Stool survey #1 was conducted during October and November 2022, a period with ongoing environmental poliovirus detection and infectious virus isolation. Survey #2, conducted from January to April 2023, finished 5 months after the last environmental isolation of poliovirus in November 2022. No poliovirus was detected over our study period, however, a range of non-poliovirus enteroviruses were detected at an incidence consistent with national enterovirus surveillance and previous reports [17,18], indicating that the overall study design and laboratory approach was valid and would have detected poliovirus if present [19].

By testing immediately available residual stool samples from children collected via routine clinical pathways, we were able to mount a much faster surveillance response using available staff resources, without the need to establish new clinical sampling pathways. The study attempted to detect sub-clinical poliovirus infection, therefore children were not selected based on suspicion of enterovirus infection. Samples tested were from children who required a stool test following a clinical evaluation within the catchment area of the hospital group, usually for investigation of illness of any type, including sepsis or febrile episodes associated with gastrointestinal disturbance. Our cohort included children from all the largest ethnic groups in the local area, and all ages up to 16 years. The largest number of samples were from children under 5 years and of Asian and Asian British ethnic group. Vaccination status, which was not confirmed at the time of sample collection, was subsequently available for > 90% of the children sampled. The vaccination rate among the children sampled was unexpectedly higher than the average for both the local area, taken as the surrounding local authorities for the hospital catchment area, and for Greater London.

As poliovirus is both potentially clinically severe and a public health risk, it was agreed, with the approval of the ethics committee, samples would not be anonymised and that results would be reported back for clinical consideration and immediate public health follow-up if appropriate. The opportunistic design required that samples be tested rapidly for poliovirus without explicit consent, without anonymisation, while allowing routine clinical diagnostic results to be provided to clinical teams. This way, the child and their parents, their doctors and health protection teams could have been informed had poliovirus been detected as part of the study.

These community residual stool surveys did not detect any poliovirus. The observations of the study and the feasibility of its pragmatic design provide important context for the ongoing transmission of VDPV observed through environmental surveillance. Given the demographic of the cohort, it is probable most children would have received IPV and therefore could still become infected and spread poliovirus without exhibiting any symptoms, as IPV provides less protection against gastrointestinal replication of poliovirus [20]. These data provide additional evidence that infection was not widespread in areas of environmental poliovirus isolation. The area of London sampled in this study is a very ethnically and socioeconomically diverse part of the UK [21,22]. Within this area there are known groups with low vaccination coverage with links to communities outside the UK that still use OPV [22,23]. While the study included children of all ethnicities, these were not in proportion to the distribution of ethnic groups across the four London Boroughs that include the catchment areas of wastewater detection. A possible explanation of our results is that any poliovirus transmission was limited to specific under-vaccinated communities and/or groups with links to populations with continuing OPV use, which may not have been well sampled in our study. Subsequent studies have shown that the poliovirus environmental detections in London were genetically linked to a clinical case of paralytic polio in the US and environmental detections in Israel [9,10]. The fact that our cohort had been sampled for clinical investigation (without suspicion of poliovirus infection), following presentation to medical services, suggests the sampled children are engaged with healthcare.

We could not investigate socioeconomic representativeness as socioeconomic data was not available for the individuals sampled. The data from children included in this study showed a high rate of primary vaccination and booster vaccination, although the vaccination data do not always record vaccinations given outside the UK. It is likely that the study was poorly targeted to the populations at highest risk of poliovirus infection: those with no or few vaccinations as part of an under-vaccinated, susceptible cohort where virus transmission can be sustained due to reduced population immunity.

Inclusion of participants based on unvaccinated status might improve ascertainment of the children with highest susceptibility in future studies, although this requires rapid access to vaccination status, which is currently not always feasible in London. However, this study design, or a refined version of it, provides a template for real time investigation in the context of prolonged environmental poliovirus surveillance detection without clinical cases of polio, as has now occurred in several European countries.

## Conclusion

The residual stool survey design presented here meets the WHO recommendations for response to poliovirus events, and is a model for rapidly enhanced poliovirus surveillance, with inherent limitations from untargeted sampling that does not capture under-vaccinated groups. While our study shows that there was no widespread poliovirus infection across the local areas of environmental detection, it is unlikely to have adequately captured populations that were at highest risk for infection. Future study designs with more resource allocation could be improved by also considering specific at-risk groups, with links to international communities where OPV is used, within the environmental surveillance catchment areas where poliovirus has been detected.

## Supplementary Data

134000 Supplement
