# Supplementary material for: Community surveillance after detection of poliovirus in the environment in London, United Kingdom, October 2022 to April 2023
Source: Euro Surveill. 2025 Apr 24;30(16):2500025. doi: 10.2807/1560-7917.ES.2025.30.16.2500025 (PMC12023727; doi:10.2807/1560-7917.ES.2025.30.16.2500025)
Supplement: Supplement [file 25-00025_ROWLAND_Supplement.pdf]

This supplementary material is hosted by *Eurosurveillance* as supporting information alongside the article "*Community surveillance after detection of poliovirus in the environment in London, United Kingdom, October 2022 to April 2023*", on behalf of the authors, who remain responsible for the accuracy and appropriateness of the content. The same standards for ethics, copyright, attributions and permissions as for the article apply. Supplements are not edited by *Eurosurveillance* and the journal is not responsible for the maintenance of any links or email addresses provided therein.

Supplementary Methods

Sample collection

The Barts hospital group comprises 5 hospitals (St Bartholomews, Newham, Whipps Cross, Royal London and Homerton in North East London, serving a population base of approximately 1.2 million of all ages, in Hackney, Newham, Tower Hamlets and Waltham Forest local authority areas in Greater London. Children resident in this area, presenting with mild illness to primary care General Practitioner (GP) services or as part of emergency admissions, who submit clinical samples, are covered by NHS pathology service arrangements, which also cater for local GPs in the same areas. All clinical samples taken for microbiological investigation for all of the five hospitals and local GPs are consolidated to a single point on a daily basis. The existing operational infrastructure allowed residual stool samples (range 1-40 samples/day) to be tested for enteroviruses and polio viruses after the normal diagnostic process. The stool samples were submitted as part of ongoing clinical care, and not specifically for study purposes.

Molecular Characterisation & Virus Isolation

Stoolsurvey#1 Residual stool materials were prepared into a 10% (w/v) faecal suspension and transported in a daily batch to Manchester UKHSA laboratory (Figure 3). Enterovirus detection was performed by rRT-PCR as previously described (1)with modifications. Briefly, following nucleic acid extraction of faecal extracts enterovirus detection was performed using primers EVF 5'GCCCCTGAATGCGGCTAAT3', EVR 5'AAACACGGACACCCAAAGTA3' and probe EVPr

5'FAM-TCTGYRGCGGAACCGACT-MGB 3', with the following cycling conditions: 15 min at 50 °C, 95 °C for 2 min followed by 45 cycles at 95 °C for 15 s and 60 °C for 1 min. Genotype assignment was achieved by partial amplification of VP1 gene and subsequent sequencing, as previously described (2).

Stool survey#2. Daily residual stool survey samples were sent to Polio Reference laboratory at UKHSA Colindale for polio virus isolation, in addition to the preparation of a 10% (w/v) faecal suspension and testing as above (survey #1). Virus isolation in cell cultures was as specified by the WHO polio surveillance protocol performed according to WHO recommended method (3–5).

Briefly, untreated faecal material (average sample weight 1.52g +2.88/-1.52) was resuspended in buffered saline and extracted with chloroform (10%(v/v)) (2x) to release bound polio and non-polio enterovirus and remove bacterial and membrane-bound virus infectivity. Aliqoutsof aqueous extract were inoculated into both Rhabdomyosarcoma (RD) and mouse L20B cells expressing the human PV receptor, cells sensitive to a broad range of polio and non-polio enteroviruses and specific for polioviruses, respectively. Cells were grown in 25cm^2 cell culture flasks and approximately 10% of the total of each extract was analysed in each cell type. Cells were examined microscopically for cytopathic effect (CPE) for up to 5 days post inoculation at which point negative cultures were blind passed into the same cells or through a cross passage strategy between RD and L20Bs confirm polio or non-polio enteroviral identity. At the end of the incubation period cell cultures were freeze thawed and aliquots from each tested by the pan-enterovirus and pan-poliovirus PCR assays from the WHO intratypic differentiation assay suite (6).

All enterovirus positive samples were sequenced using Oxford Nanopore Technologies protocols following protocols previously described (7). This is a nested pan-enterovirus/polio specific product assay with a bespoke polio bioinformatic analysis module, but since no polioviruses were detected in this study the first round pan-enterovirus products were

sequenced and typed by comparison with GenBank database sequences to confirm enterovirus isolate identities.
